# Broad phenotypic spectrum and genotype-phenotype correlations in *GMPPB*-related dystroglycanopathies: an Italian cross-sectional study

**DOI:** 10.1186/s13023-018-0863-x

**Published:** 2018-09-26

**Authors:** Guja Astrea, Alessandro Romano, Corrado Angelini, Carlo Giuseppe Antozzi, Rita Barresi, Roberta Battini, Carla Battisti, Enrico Bertini, Claudio Bruno, Denise Cassandrini, Marina Fanin, Fabiana Fattori, Chiara Fiorillo, Renzo Guerrini, Lorenzo Maggi, Eugenio Mercuri, Federica Morani, Marina Mora, Francesca Moro, Ilaria Pezzini, Esther Picillo, Michele Pinelli, Luisa Politano, Anna Rubegni, Walter Sanseverino, Marco Savarese, Pasquale Striano, Annalaura Torella, Carlo Pietro Trevisan, Rosanna Trovato, Irina Zaraieva, Francesco Muntoni, Vincenzo Nigro, Adele D’Amico, Filippo M. Santorelli, the Italian CMD Network, Angela Berardinelli, Giacomo Comi, Maria Alice Donati, Maria Teresa Dotti, Marina Grandis, Francesca Magri, Maria A. Maioli, Alessandro Malandrini, Francesco Mari, Roberto Massa, Luciano Merlini, Maurizio Moggio, Lucia O. Morandi, Olimpia Musumeci, Marika Pane, Antonella Pini, Elena Pegoraro, Elena M. Pennisi, Lorenzo Peverelli, Giulia Ricci, Carmelo Rodolico, Lucia Ruggiero, Michele Sacchini, Lucio Santoro, Gabriele Siciliano, Alessandro Simonati, Paola Tonin, Antonio Toscano

**Affiliations:** 1 Department of Developmental Neuroscience and Molecular Medicine Neuromuscular Unit and Child Neurology, IRCCS Fondazione Stella Maris, Via dei Giacinti 2, 56018 Pisa, Italy; 2 Neuropathology Unit, Institute of Experimental Neurology and Division of Neuroscience, IRCCS San Raffaele Scientific Institute, Milan, Italy; 3 Fondazione San Camillo Hospital IRCCS, Lido Venice, Italy; 4 Department of Neuroimmunology and Neuromuscular Disorders, Neurological Institute “C. Besta” IRCCS Foundation, Milan, Italy; 5 Rare Diseases Advisory Group Service for Neuromuscular Diseases, Muscle Immunoanalysis Unit, Dental Hospital, and The John Walton Muscular Dystrophy Research Centre, MRC Centre for Neuromuscular Diseases Institute of Genetic Medicine, University of Newcastle, Newcastle upon Tyne, UK; 6 Department of Clinical and Experimental Medicine, University of Pisa, Pisa, Italy; 7 Department of Medical, Surgical and Neurosciences, University of Siena, Siena, Italy; 8 Unit of Neuromuscular and Neurodegenerative Disorders, Department of Neurosciences, Bambino Gesù Children’s Hospital, Rome, Italy; 9 Center of Myology and Neurodegenerative Disorders, G. Gaslini Institute, Genoa, Italy; 10 Neurological Science Department and Venetian Institute of Molecular Medicine, University of Padua, Padua, Italy; 11 Pediatric Neurology and Muscular Diseases Unit, Department of Neurosciences, Rehabilitation, Ophthalmology, Genetics, Maternal and Child Health, University of Genoa, “G. Gaslini” Institute, Genoa, Italy; 12 Pediatric Neurology Unit and Laboratories, Children’s Hospital A. Meyer-University of Florence, Florence, Italy; 13 Pediatric Neurology Unit, Department of Women’s and Children’s Health, Università Cattolica del Sacro Cuore, Rome, Italy; 14 Cardiomyology and Genetic Section, Department of Internal and Experimental Medicine, University of Campania “Luigi Vanvitelli”, Naples, Italy; 15 Department of Translational Medicine, Federico II University, Naples, Italy; 16 Telethon Institute of Genetics and Medicine, Pozzuoli, Naples Italy; 17 Sequentia Biotech SL, Barcelona, Spain; 18 Dipartimento di Biochimica, Biofisica e Patologia Generale, Università degli Studi della Campania “Luigi Vanvitelli”, Naples, Italy; 19 Folkhälsan Institute of Genetics, Haartman Institute, University of Helsinki, Helsinki, Finland; 20 Department of Neurological and Psychiatric Sciences, University of Padua, Padua, Italy; 21 Dubowitz Neuromuscular Centre (F. Muntoni), UCL Great Ormond Street Institute of Child Health, London, UK; 22 NIHR Great Ormond Street Hospital Biomedical Research Centre, 30 Guilford Street, London, WC1N 1EH UK

**Keywords:** Congenital muscular dystrophy, Limb-girdle muscular dystrophy, *GMPPB*, Dystroglycanopathies, Genotype-phenotype correlations

## Abstract

**Background:**

Dystroglycanopathy (α-DG) is a relatively common, clinically and genetically heterogeneous category of congenital forms of muscular dystrophy (CMD) and limb-girdle muscular dystrophy (LGMD) associated with hypoglycosylated α-dystroglycan. To date, mutations in at least 19 genes have been associated with α-DG. One of them, *GMPPB*, encoding the guanosine-diphosphate-mannose (GDP-mannose) pyrophosphorylase B protein, has recently been associated with a wide clinical spectrum ranging from severe Walker-Warburg syndrome to pseudo-metabolic myopathy and even congenital myasthenic syndromes.

We re-sequenced the full set of known disease genes in 73 Italian patients with evidence of either reduced or nearly absent α-dystroglycan to assess genotype-phenotype correlations in this cohort. We used innovative bioinformatic tools to calculate the effects of all described *GMPPB* mutations on protein function and attempted to correlate them with phenotypic expressions.

**Results:**

We identified 13 additional cases from 12 families and defined seven novel mutations. Patients displayed variable phenotypes including less typical pictures, ranging from asymptomatic hyperCKemia, to arthrogryposis and congenital clubfoot at birth, and also showed neurodevelopmental comorbidities, such as seizures and ataxic gait, as well as autism-spectrum disorder, which is seldom described in clinical reports of dystroglycanopathies. We also demonstrated that few mutations recur in the Italian *GMPPB*-mutated population and that alterations of protein stability are the main effects of *GMPPB* missense variants.

**Conclusion:**

This work adds to the data on genotype-phenotype correlations in α-DG and offers new bionformatic tools to provide the conceptual framework needed to understand the complexity of these disorders.

**Electronic supplementary material:**

The online version of this article (10.1186/s13023-018-0863-x) contains supplementary material, which is available to authorized users.

## Background

Muscular dystrophies with evidence of reduced glycosylation of the transmembrane glycoprotein α-dystroglycan on skeletal muscle biopsy [1] are collectively termed α-dystroglycanopathy (α-DG) [2–4], and they constitute a clinically and genetically heterogeneous group of autosomal recessive muscular dystrophies with variable neurological and ophthalmic involvement.

The phenotypic severity of α-DG patients is extremely variable. At the most severe end of the clinical spectrum we find Walker-Warburg syndrome (WWS), muscle-eye-brain disease and Fukuyama congenital muscular dystrophy. These conditions are characterized by congenital muscular dystrophy (CMD) and severe structural brain and eye abnormalities, leading to early infantile death in WWS [5]. Conversely, individuals at the mildest end of the clinical spectrum may present, sometimes in adulthood, with limb-girdle muscular dystrophy (LGMD), and without associated brain or eye involvement [6].

Mutations in six genes (*POMT1*, *POMT2*, *POGnT1*, *FKRP*, *FKTN*, and *LARGE*) are by far the most common in large Italian and UK cohorts ascertained by low skeletal muscle expression of α-dystroglycan [7, 8], but they account for only about 50% of cases, leaving the rest without a molecular diagnosis. The advent of next-generation sequencing (NGS) methodologies has rapidly expanded the number of α-DG-related genes and subsequently resulted in an expansion of the clinical spectrum observed in affected children and adults [9, 10].

To date, mutations in 19 genes coding for dystroglycan itself or more frequently, for glycosyltransferases and accessory proteins involved in the post-translational modification of α-dystroglycan, are documented to be responsible for the different forms of α-DG. One of them, *GMPPB*, coding for the guanosine-diphosphate-mannose (GDP-mannose) pyrophosphorylase B protein, seems to be particularly frequent and is associated with a wide spectrum of muscle weakness, ranging from WWS to a mild form of adult-onset LGMD overlapping with different congenital myasthenic syndromes (CMSs) [11–13]. More recently pseudo-metabolic features have been described in few patients [14, 15]. Overall, about 81 *GMPPB*-mutated patients have been described worldwide: 56 with LGMD or overlapping LGMD-CMS phenotypes and the rest presenting features of CMD. To assess the relative frequency of *GMPPB* variants and contribute to the definition of the associated clinical manifestations, we systematically screened a large Italian population of α-DG patients for mutations. We used new bioinformatics tools to assess how mutations found in this study may affect protein function. We then combined the data from available families to determine mutation frequencies in relation to clinical severity, and thus establish more precise genotype-phenotype correlations.

## Methods

This study was approved by the ethics committees of our institutions. The patients were recruited after written informed consent that had been obtained in accordance with national regulations.

All the cases are part of a multicenter study that aims to improve molecular characterization of presently undefined forms of CMD associated with defective glycosylation of α-dystroglycan. For the present work, we collected all genetically undefined patients with low α-dystroglycan levels currently followed at any of the tertiary care centers for pediatric and adult neuromuscular disorders belonging to the Italian CMD network. Cases with milder phenotypes, and a possible diagnosis of LGMD or LGMD-CMS, were also investigated.

We selected, from the overall network population, 105 patients with muscle biopsy-confirmed low α-dystroglycan expression. Prior to our molecular investigations, two expert co-authors (C.F., A.R.) blindly reviewed these patients’ histological and immunohistochemical features. On the basis of their findings, 32 patients, with a reduction of α-dystroglycan likely unrelated to glycosylation defects, or no reduction at all, were excluded. Thus, our study included 73 patients fully meeting the diagnostic criteria for dystroglycanopathy described elsewhere [9].

In this work, past medical history and clinical information, as well as neurophysiological, brain MRI and myoimaging (available only in two patients) are described for the 13 patients in whom we identified bi-allelic mutations in *GMPPB.* Patients who showed a significant delay in motor abilities and late acquisition of walking (> 24 months) were classified as affected by CMD, whereas those in whom overt clinical manifestations appeared later, as described elsewhere [16], were deemed to present with LGMD. Clinical subcategories of CMD were defined as others have done [17]. Whenever possible patients have since been reassessed and submitted to a detailed clinical re-examination.

Molecular genetic analyses were carried out in all 73 patients and, when available, in their parents and siblings. Genomic DNA was purified from whole blood using standard methodologies and the coding regions of 93 genes linked to CMD, LGMD or related diseases were investigated in a single tube using Dystroplex, an extended NGS testing panel covering at high depth the tested genes and described elsewhere [15, 18]. In all the cases, sequencing was performed with Illumina technology and standard bioinformatics pipelines were applied for quality control, mapping, variant calling and annotation. Publicly available databases (HGMD: http://www.hgmd.cf.ac.uk/ac/index.php; LOVD: http://www.dmd.nl/; 1000G database: http://www.1000genomes.org/; dbSNP database: http://www.ncbi.nlm.nih.gov/SNP/; Sequencing Initiative Suomi (SISu): www.sisuproject.fi; gnomAD; http://gnomad.broadinstitute.org/; ExAC: http://exac.broadinstitute.org/ and EVS: http://evs.gs.washington.edu/EVS/) (last access December 2017) were interrogated to identify previously reported variants and also to determine the frequency of the novel variants observed. The pathogenicity assessment of the target variant was performed according to the guidelines published by the American College of Medical Genetics for the interpretation of sequence variants [19]. Standard in silico tools (Polyphen: http://genetics.bwh.harvard.edu/pph2/; SIFT: sift.jcvi.org/; UMD-Predictor: http://umd-predictor.eu/analysis.php and Mutation taster: http://www.mutationtaster.org/) were used to assess the deleteriousness of missense mutations.

A three-dimensional model of human *GMPPB* was obtained using a combination of threading and homology modeling methods [20]. The quality of predicted models was assessed using the Qmean server [21] and energy minimization of protein models was carried out using the 3DRefine web server [22]. The effects of *GMPPB* missense mutations on protein stability and the change in thermodynamic folding stability (the difference in Gibbs free energy between wild-type and mutant, ΔΔG) were calculated using FoldX [23], an algorithm that uses an empirical force field to evaluate the effect of mutations on protein stability. The calculated free energy differences (ΔΔG) [24] indicate the change in structural stability, with negative values indicating amino acid substitutions that tend to increase the thermodynamic structural rigidity of the proteins, and positive values indicating variants that tend to destabilize proteins. The RepairPDB function was applied to the *GMPPB* wild-type structure before running the BuildModel function of FoldX, and nine independent runs were carried out for each mutation. The prediction error of FoldX is approximately 0.5 kcal/mol, therefore changes in that range are insignificant. UCSF Chimera software (version 1.11) [25] was used for molecular graphics and GraphPad Prism was used for data analysis and curve fitting.

Western blotting (WB) was undertaken as described [26] on a limited number of samples (P1, P2, P3, P6, and P7), depending on material availability. Mouse monoclonal antibodies used on blots were to β-dystroglycan (NCL-b-*DG, Leica Biosystems) and* laminin α2 (MAb 1922, Chemicon). Western blots were visualized using the Pierce Supersignal detection system according to the manufacturer’s directions. The density of the myosin heavy chain (MHC) band on the Coomassie blue stained, post-blotted gel was a marker for protein loading.

## Results

Thirteen patients (8 men and 5 women, age range at last examination 20 months–74 years) from a cohort of 73 Italian α-DG cases showed two predictably pathogenic mutations in *GMPPB.* The patients’ clinical data are summarized in Table 1. Patient P6 has already been described in detail elsewhere [15]. Patients 9 and 10 are uncle and nephew; the remaining patients are unrelated.

**Table 1:** Clinical features in 13 patients harboring bi-allelic mutations in *GMPPB*

Case/Sex	P1/M	P2/M	P3/M	P4/F	P5/M	P6/F	P7/M	P8/M	P9/M^a^	P10/M^a^	P11/F	P12/F	P13/M
Mutations	p.I219T p.R287Q	p.T153I p.Q234^*^	p.K31E p.G174S	p.T153I p.P32L	p.P32L p. R287Q	p.G315S p.D27H	p.S168F p.Q234^*^	p.R243W p.P32L	p.D27H p.V330I	p.D27H p.V330I	p.R288Q p.C285YfsX19	p.G220R p.R287Q	p.R287Q p.V330I
Diagnosis	CRB	MEB	CMD-MR	CRB	CRB	LGMD	LGMD	LGMD	LGMD	LGMD	LGMD	LGMD	LGMD
Onset	18 months	birth	birth	birth	birth	6 years	13 years	20 years	28 years	24 years	7 years	25 years	34 years
Distribution of weakness	Axial and upper girdle	Proximal lower girdle	generalized	generalized	generalized	Mild upper and lower limb girdle	Mild proximal	Mild proximal	Mild upper and lower limb girdle	Mild upper and lower limb girdle	Mild proximal	Mild proximal	Mild proximal
Maximal motor acquisition	Independent walk with widening base	Independent walk with flat foot	Sitting position	none	none	Run with mild clumsiness	run	run	Run but loss of ambulation at 58 years	run	run	run	run
Intellectual disability	+ (mild) ASD	+ (mild)	+ (mild)	+ (severe)	+ (severe)	–	–	–	–	–	–	–	–
Epilepsy	+	+	+	+ drug resistant	+	+	–	–	–	–	–	–	–
Brain MRI findings: Cerebellar hypoplasia WMC GMC	mild - Microcephaly	- + -	- - Microcephaly	mild - Polymicrogyria/Microcephaly	mild - Cortical hypoplasia/Microcephay	- + -	Not performed	Not performed	Not performed	Not performed	Not performed	Not performed	Not performed
Ophthalmologic findings	Strabismus Nystagmus Cataract	Congenital cataract	–	Congenital cataract	–	–	–	–	–	–	–	–	–
Cardiorespiratory findings	–	Respiratory distress at birth	Respiratory distress at birth	Respiratory distress at birth, sudden heart block at 20 months	–	Wolff-Parkinson-White type B syndr.ome	–	–	–	–	Short QT interval	–	–
Other features	–	Facial dimorphisms	Facial dimorphisms, foot deformities kyphosis	Arthrogryposis	Tube Feed	Exercise intolerance myoglobinuria	Exercise intolerance	Easy fatigability	–	–	Scoliosis fatigability	Scoliosis fatigability	Mild fatigability

Abbreviations: *M* men, *F* women, *WMC* white matter changes, *GMC* grey matter changes, *CRB* congenital muscular dystrophy with cerebellar involvement, *CMD-MR* congenital muscular dystrophy with mental retardation, *LGMD* limb girdle muscular dystrophy;+ present; − absent; ^a^these patients are uncle and nephew; ASD autism spectrum disorder; MEB, muscle-eye-brain disease

*indicates in genetics the presence of a STOP CODON

Five patients (P1–P5) presented onset at birth or in the first year of life, and three (P6, P7, P11) during childhood. The other five had adult onset of their muscle weakness (> 18 years). All 13 patients, albeit to different degrees, presented proximal weakness both of the shoulder and the pelvic girdle. Contractures and scoliosis were part of the picture in 4/13 cases, and two patients with congenital onset (P3 and P4) displayed arthrogryposis. Generalized or focal epilepsy (both tonic-clonic and focal seizures with impaired awareness and oromasticatory automatisms) was seen in all the patients with congenital onset, and in one case with childhood onset (P6). All the patients with congenital onset showed intellectual disability and were unable to produce full sentences; P1 has a diagnosis of autism spectrum disorder. Two patients with congenital onset (P2 and P4) showed bilateral cataracts. One case (P1) displayed nystagmus and inward strabismus, with reduction of lateral movement, and upgaze palsy. Two of the LGMD patients (P6 and P7) displayed exercise intolerance and four (P8, P11, P12, P13) easy fatigability; anyway only P8 received pharmacological treatment showing a partial response to pyridostigmine. No respiratory or cardiac involvement was found in this cohort, except for slight heart conduction impairment in three patients (one of whom [P6] carried a diagnosis of Wolf–Parkinson–White syndrome).

The disease was usually progressive over time, however most of the patients who achieved ambulation were still ambulant at the time of diagnosis.

Serum CK levels were increased (ranging from 316 to 38,650 UI/L); muscle biopsy showed features of a muscular dystrophy with abnormal variation in fiber size, necrosis and fibrosis, and in two cases (P5 and P6) it highlighted a myolitic process. Immunohistochemistry with the IIH6 antibody revealed variable degrees of hypoglycosylation of α-dystroglycan. Electromyography was normal in some cases, and otherwise showed mild to moderate myopathic changes. Repetitive nerve stimulation was performed in three LGMD patients (P6, P8 and P13), and only in P8 revealed findings consistent with abnormal neuromuscular transmission in proximal muscles. Brain MRI was available in 6 patients (P1-P6) and showed features previously observed in published *GMPPB*-mutated patients (Table 1). Muscle MRI was available only in two patients (P1 and P6): the first one, with a CMD phenotype, presented a prevalent involvement of vastus lateralis at thigh level and minimal involvement of soleus at the calves. The second patient with a LGMD phenotype showed minimal involvement of adductor magnus, semimebranosus, semitendinosus and sartorius muscles at thigh level, whereas revealed a mild fatty streaking in soleus and peroneal muscles at calf level.

**Fig. 1:**
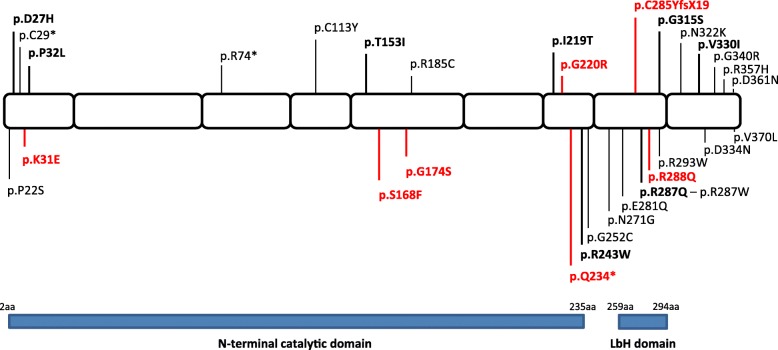
Morbidity map of *GMPPB* in Italian patients with α-dystroglycanopathy compared to mutations reported in literature. The scheme of the *GMPPB* protein is illustrated and the mutations identified in this study depicted in bold (novel mutations are in red, published mutations are in black)

**Fig. 2:**
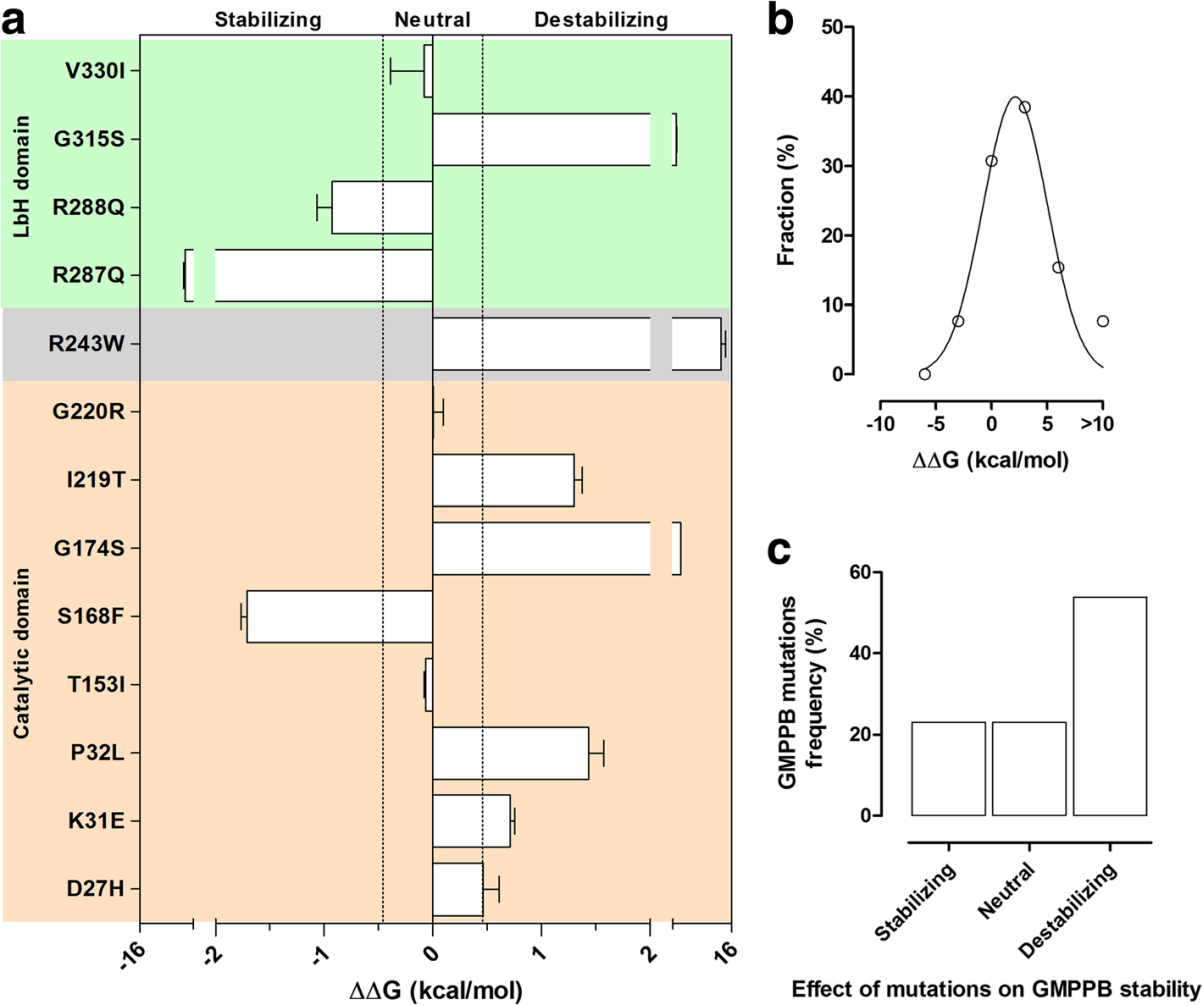
Energy changes of missense mutations in *GMPPB*. **a** Histogram of calculated free energy changes (ΔΔG) due to missense mutations in *GMPPB*. Orange-, gray- and green-shaded bars indicate mutated residues falling in the N-terminal catalytic domain, inter-domains and C-terminal LbH domain, respectively. **b** The ΔΔG distributions of *GMPPB* missense mutations. The ΔΔG values of mutations were presented in histograms, using 3 kcal/mol bins (the single mutation with ΔΔG = 13.7 kcal/mol was classified into the > 10 kcal/mol bin) and the distribution was fitted to a Gaussian function. **c** Histogram of the number of mutations (%) plotted against the predicted effect of mutations on GMPPB stability

The 13 patients carried 15 different mutations (seven were novel) in *GMPPB*, including 13 missense variants, one nonsense, and one frameshift (Fig. 1; Table 2). The mutations appear evenly distributed throughout the different domains/inter-domains of the protein and induce distinct alterations in the conformation of the protein (Additional file 1: Figure S1). The predicted ΔΔG values of the different *GMPPB* mutations were found to range from 5.7 to 13.7 kcal/mol (Fig. 2a), and showed the classical Gaussian distribution previously described for other proteins [24]. The mean of the high destabilizing score (μ = 2.1 kcal/mol, *R* = 0.96; Fig. 2b) suggested that most *GMPPB* mutations affect the thermodynamic stability of the protein. In particular, 33% of mutations were recognized as stabilizing (ΔΔG < − 0.46 kcal/mol) and 54% as destabilizing (ΔΔG > 0.46 kcal/mol) (Fig. 2c), while a small percentage appeared to be neutral (− 0.46 < ΔΔG < 0.46 kcal/mol; Fig. 2a, c).

**Table 2:** Genetic findings in patients with mutations in *GMPPB*

	Mutation	SIFT (score)	Polyphen 2 score	Protein domain	Amino acid conservation	Reference
P1	p.I219T p.R287Q	Deleterious (0.04) Tolerated (0.27)	Benign 0,267 Benign 0,117	N-TC domain LbH	moderately conserved weakly conserved	[28] [11]
P2	p.T153I p.Q234*	Tolerated (0.22) na	Possibly damaging 0,812 na	N-TC domain na	weakly conserved weakly conserved	[14] **This work**
P3	**p.K31E** **p.G174S**	Deleterious (0.02) Deleterious (0)	Probably damaging 0,992 Probably damaging 1,00	N-TC domain N-TC domain	highly conserved highly conserved	**This work** **This work**
P4	p.T153I p.P32L	Tolerated (0.22) Deleterious (0)	Possibly damaging 0,812 Probably damaging 1,00	N-TC domain N-TC domain	weakly conserved highly conserved	[14] [11]
P5	p.P32L p.R287Q	Deleterious (0) Tolerated (0.27)	Probably damaging 1,00 Benign 0,117	N-TC domain LbH	highly conserved weakly conserved	[11] [11]
P6	p.G315S p.D27H	Deleterious (0,01) Deleterious (0,02)	Probably damaging 0,989 Benign 0,089	LbH N-TC domain	highly conserved weakly conserved	[15] [11]
P7	**p.S168F** p.Q234*	Deleterious (0,01) na	Probably damaging 1,00 na	N-TC domain na	highly conserved weakly conserved	**This work** **This work**
P8	p.R243W p.P32L	Deleterious (0,02) Deleterious (0)	Benign (0,006) Probably damaging 1,00	na N-TC domain	highly conserved highly conserved	[26] [11]
P9	p.D27H p.V330I	Deleterious (0,02) Deleterious (0)	Benign 0,089 Probably damaging (0,981)	N-TC domain na	weakly conserved	[11] [27]
P10	p.D27H p.V330I	Deleterious (0,02) Deleterious (0)	Benign 0,089 Probably damaging (0,981)	N-TC domain na	weakly conserved highly conserved	[11] [27]
P11	**p.R288G** **p.C285YfsX19**	Deleterious (0.01) na	Probably damaging (0.980)	LbH LbH	moderately conserved weakly conserved	**This work** **This work**
P12	**p.G220R** p.R287Q	Deleterious (0) Tolerated (0.27)	Probably damaging (0.999) Benign 0,117	N-TC domain LbH	highly conserved weakly conserved	**This work** [11]
P13	p.R287Q p.V330I	Tolerated (0.27) Damaging(0)	Benign 0,117 Probably damaging (0,981)	LbH na	weakly conserved highly conserved	[11] [27]

Legend: *na* not applicable, *N-TC domain* N terminal catalytic domain, *LbH* Left-handed parallel beta-Helix domain. Novel mutations are in bold

*indicates in genetics the presence of a STOP CODON

**Fig. 3:**
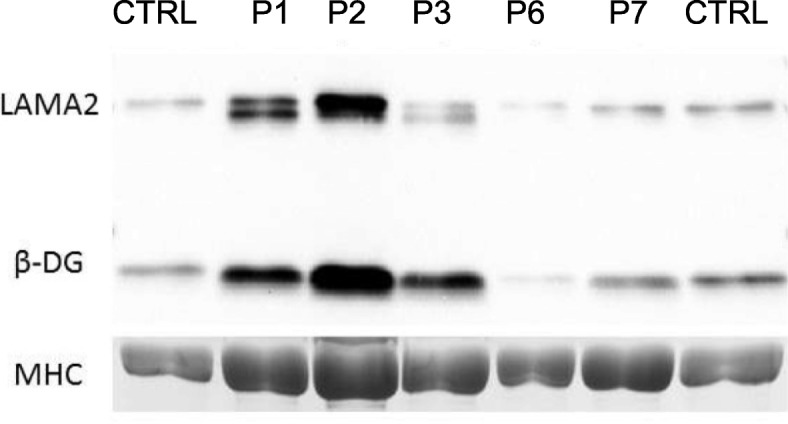
Western blot analysis of patients with mutations in the *GMPPB* gene. Consistent lower mobility shift of β-dystroglycan (β-DG) and variable expression of laminin α2 (LAMA2) in skeletal muscle biopsies from five patients (P1, P2, P3, P6, and P7) with mutations in the *GMPPB* gene. Myosin heavy chain (MHC) was used as measure of protein loading. CTRL, control muscle

Finally, we investigated whether mutations with different scores may have different effect on other proteins known to be secondarily affected in *GMPPB* dystroglycanopathy [26]. WB confirmed a mobility shift of β-dystroglycan in all the patients analyzed and a variable reduction of laminin α2, which was not correlated with the nature of mutations in *GMPPB* (Fig. 3).

## Discussion

This is the first Italian population study regarding *GMPPB-*related dystroglycanopathy and encompassing all the previously reported related clinical phenotypes. Only 18% of the patients in our cohort (13/73) harbored pathogenic mutations in *GMPPB*, and only five presented a CMD phenotype. This latter finding is in line with data from the literature [11, 26–28], which indicate that mutations in *GMPPB* are more common in relatively milder forms of neuromuscular disorders.

No significant clinical findings seemed to emerge in the CMD group, as the features found in these patients, including intellectual disability, ophthalmic involvement, epilepsy and microcephaly, are also typical of other dystroglycanopathies [11]. However, what our study adds to this already broad clinical spectrum is the possible presence of arthrogryposis and congenital clubfoot, particularly in patients with very severe, generalized involvement, as well as nystagmus and upgaze palsy.

Intellectual disability was evident in all the congenital forms, predominantly affecting the language domain. Epilepsy appeared to be related to cognitive impairment and not to the presence of MRI alterations. Autism spectrum disorder emerged as a rare neurodevelopmental comorbidity. At this stage, we cannot totally exclude that an additional variant in neurodevelopmental genes can co-occur, at least in some patients, in a sort of “double trouble” condition. Contrary to the findings of others [14], none of our patients displayed movement disorders such as chorea, whereas ataxia could be part of the clinical picture in line with possible evidence of cerebellar atrophy. On the basis of data from the literature and from our case studies, it can confidently be asserted that mutations in *GMPPB* predominantly affect the brain in CMD infants. Unless these patients’ clinical and laboratory features are evaluated in a tertiary center specializing in neuromuscular diseases, this might lead to a suspicion of encephalopathy and prevent the condition from being diagnosed early.

The onset of motor manifestations in the LGMD group occurred at different ages and, as previously reported, the extent of weakness was unrelated to the timing of onset of the disease. On the other hand, features such as intellectual disability or epilepsy can be the first manifestations of the disease and are frequent in those patients who have earliest (< 18 years) muscle involvement. None of our LGMD patients displayed cognitive impairment or brain MRI alterations.

The patients with the milder forms manifested easy fatigability or myoglobinuria, or (P8) presented relatively asymptomatic hyperCKemia with subtle weakness, evident only on expert clinical examination. Few cases had overlapping LGMD and CMS features, however we did not specifically set out to identify patients with pathological neurophysiological data, and abnormal neuromuscular transmission in proximal muscles was detected in P8 only after establishing the molecular diagnosis. Contrary to literature data, none of our patients showed facial weakness or ptosis and cataracts were not invariably detected in our patients.

Muscle MRI did not reveal, in our cohort, a striking pattern of muscle involvement maybe in consideration of the different ages and phenotypes displayed by the two patients analyzed (P1 and P6). However, as reported previously [13, 26] we observed a prevalent involvement of posterior compartment of the thigh with a relative atrophy of the anterior compartment, and a minor involvement of the lower limb.

Through this work, we have expanded the array of pathogenic variants associated with *GMPPB*, and shown that these mutations are widely distributed within the coding exons and located both in the C and in the N-terminal domains (see Additional file 1: Figure S1). In this sample at least, the clinical phenotype did not appear to be related to a specific mutation site within the protein structure. Nonetheless, in an attempt to identify possible genotype-phenotype correlations, we analyzed the allelic frequency of the common variants identified in our population and studied the effects of these mutations on protein stability computing the changes in the thermodynamic folding free energy (ΔΔG). Two mutations (p.R287Q and p.D27H) were found to be common in our study, having an allelic frequency of 15.4 and 11.5%, respectively, rates similar to those reported in the literature [27]. Conversely, p.V330I, found in 3/26 alleles in our study, has only occasionally been reported elsewhere. Interestingly, p.V330I appeared to be pseudo-dominantly inherited in one family (P9 and P10, Additional file 2: Figure S2). We cannot firmly establish whether family members were distantly related and the possibility of an independent inheritance of the p.D27H mutation cannot be excluded.

Examining our data we observed that all the patients carrying p.D27H (P6, P9, P10) showed a mild phenotype as previously described, and this is in keeping with this mutation’s neutral effect on protein stability (Fig. 2a). The reverse is true for p.D287Q whose ΔΔG value predicted a stabilizing effect on the protein. Furthermore, p.R287Q, if combined with the likely destabilizing effect of p. I219T or p.P32L, might predict a severe phenotype (as in P1 or P5, respectively), whereas its association with a neutral variant (e.g. p.G220R in P11 or p.V330I, as in P12) might suggest a less aggressive phenotype. Similar considerations probably apply in the case of association of the most common variant, p.D27H (often seen in LGMD-CMS patients), with the more severe p.P32L mutation.

Consistent with previous findings [26], we observed that patients with *GMPPB* dystroglycanopathy share the unique biochemical feature of a change in electrophoretic mobility of β-dystroglycan. As there was not apparent correlation between the predicted stability of mutated *GMPPB* and residual expression of glycosylated α-dystroglycan or secondary reduction of laminin α2 (Fig. 3 and not shown), the finding that β-dystroglycan is equally affected in all patients regardless of the predicted stability of the mutations suggests that the overall retained function of *GMPPB* may be key to the variability in patients’ phenotype.

## Conclusions

To summarize, this study describes a sample of 13 Italian patients carrying a total of 15 different mutations in *GMPPB*, representing 18% of our study cohort of α-DG patients. Accordingly, *GMPPB* seems to be one of the more frequent “second generation” α-DG-related genes discovered in the NGS era. Our findings, combined with literature data, show that there are at least three forms of *GMPPB*-related myopathy: i) CMD, ii) early onset LGMD, and iii) adult onset LGMD, often with evidence of neuromuscular junction involvement. Less severe phenotypes are also observed, such as exercise intolerance and myoglobinuria (in P6) or asymptomatic hyperCKemia (P8). In the absence of information on residual enzyme activity in tissues, combining clinical findings with bioinformatic data on variant stability might allow objective assessment of disease severity.

## Additional files

Additional file 1: **Figure S1.** (**A**) Effects of missense mutations on the structure of GMPPB. The images show the close-up of the different mutation sites with the predicted consequences of the amino acid replacement. Wild-type protein is shown in gray and mutated proteins in magenta. The side chain of wild-type and mutated residues are shown as sticks. Mutated residues located in the N-terminal catalytic domain, inter-domains and C-terminal LbH domain are shown on orange, gray and green backgrounds, respectively. (**B**) Distribution of missense mutations between the domains and inter-domains of the GMPPB protein reported as number of mutations per amino acid. Orange bar, N-terminal catalytic domain; gray bar, inter-domains; green bar, C-terminal LbH domain. (PDF 8212 kb)

Additional file 2: **Figure S2.** Pedigree of the family showing pseudo-dominant inheritance in GMPPB disease. Two patients (uncle and nephew, P9 and P10, respectively) showed onset in early adulthood and similar muscular impairments associated with biallelic mutations (p:Asp27His and p.Val330Ile) in *GMPPB*. Circles are females and squares are males. Slashed symbols indicate deceased individuals. Numbers in symbols indicate number of siblings. (PDF 7 kb)

## Abbreviations

CMD: Congenital muscular dystrophy
CMS: Congenital myasthenic syndrome
GMPPB: Guanosine-diphosphate-mannose (GDP-mannose) dyrophosphorylase B gene
LGMD: Limb-girdle muscular dystrophy
MRI: Magnetic resonance imaging
NGS: Next-generation sequencing
WWS: Walker-Warburg syndrome
α-DG: alpha-dystroglycanopathy
