# Supplementary figures and images for: Broad phenotypic spectrum and genotype-phenotype correlations in GMPPB-related dystroglycanopathies: an Italian cross-sectional study
Source: Orphanet J Rare Dis. 2018 Sep 26;13:170. doi: 10.1186/s13023-018-0863-x (PMC6158856; doi:10.1186/s13023-018-0863-x)

A

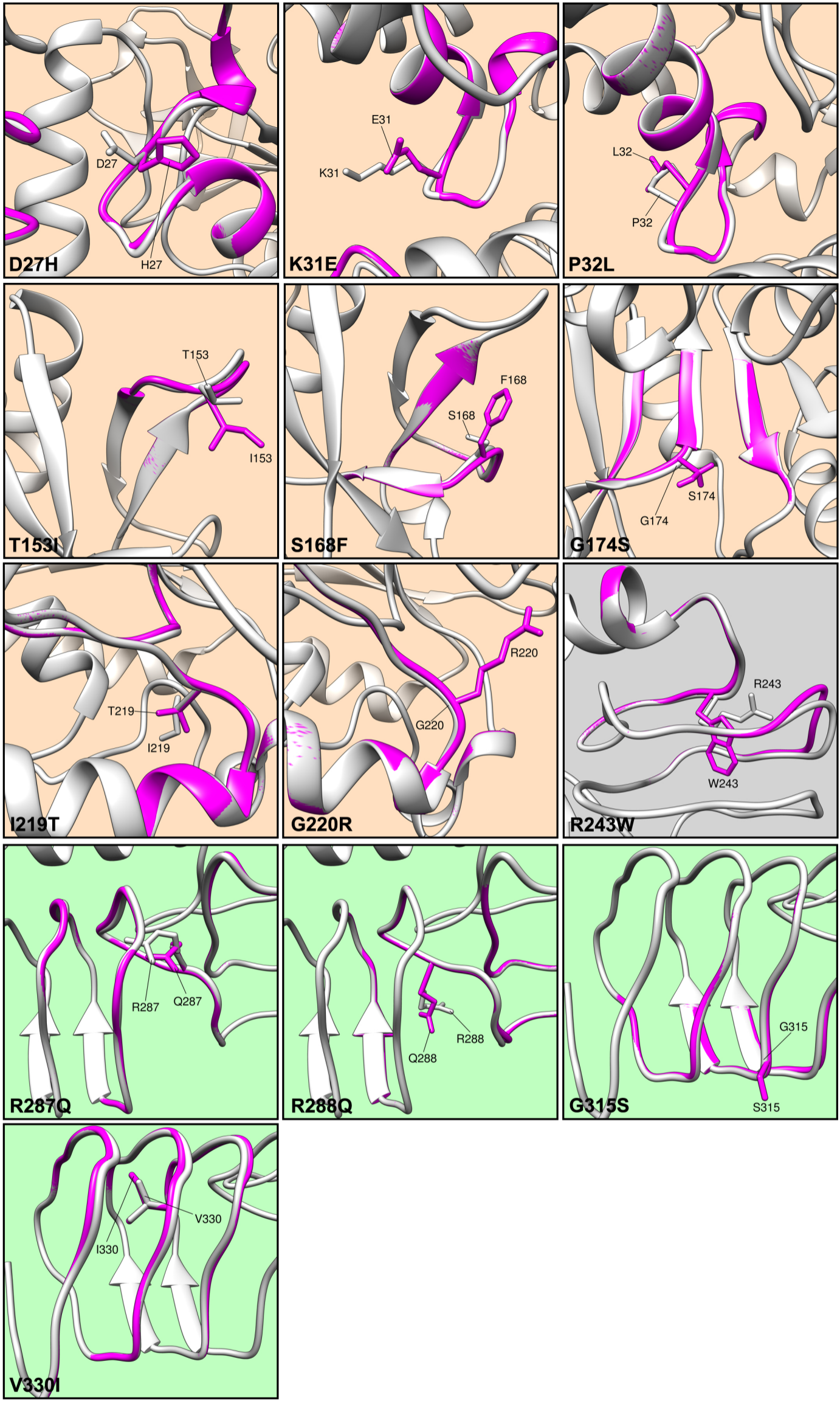

B

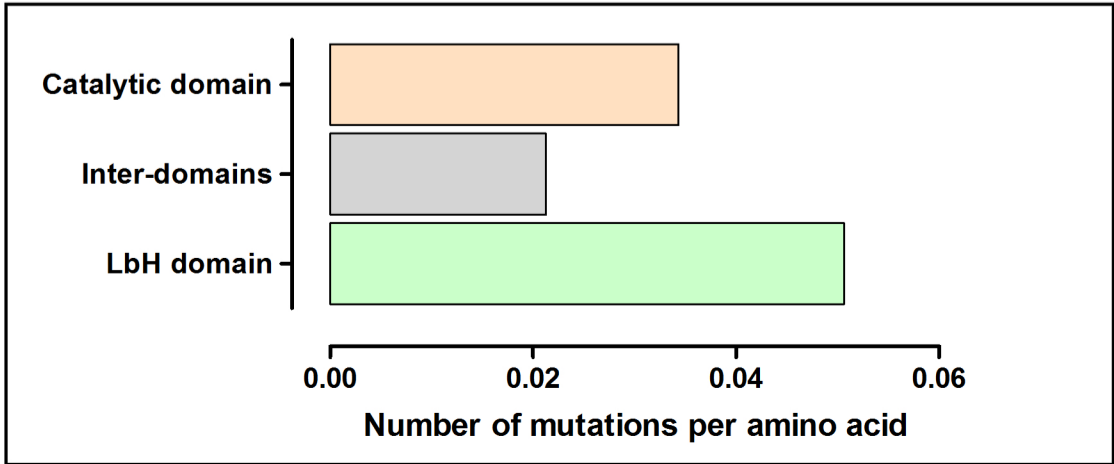

Supplement: Supplementary file 1 — Figure S1. (A) Effects of missense mutations on the structure of GMPPB. The images show the close-up of the different mutation sites with the predicted consequences of the amino acid replacement. Wild-type protein is shown in gray and mutated proteins in magenta. The side chain of wild-type and mutated residues are shown as sticks. Mutated residues located in the N-terminal catalytic domain, inter-domains and C-terminal LbH domain are shown on orange, gray and green backgrounds, respectively. (B) Distribution of missense mutations between the domains and inter-domains of the GMPPB protein reported as number of mutations per amino acid. Orange bar, N-terminal catalytic domain; gray bar, inter-domains; green bar, C-terminal LbH domain. (PDF 8212 kb) [file 13023_2018_863_MOESM1_ESM.pdf]

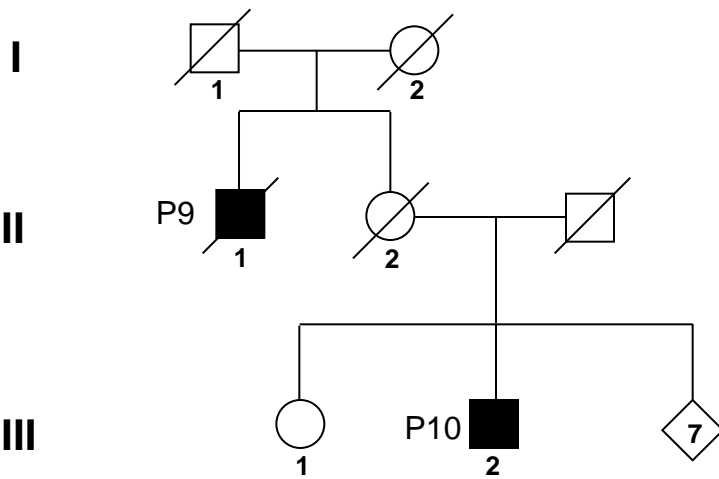

Supplement: Supplementary file 2 — Figure S2. Pedigree of the family showing pseudo-dominant inheritance in GMPPB disease. Two patients (uncle and nephew, P9 and P10, respectively) showed onset in early adulthood and similar muscular impairments associated with biallelic mutations (p:Asp27His and p.Val330Ile) in GMPPB. Circles are females and squares are males. Slashed symbols indicate deceased individuals. Numbers in symbols indicate number of siblings. (PDF 7 kb) [file 13023_2018_863_MOESM2_ESM.pdf]
